# Emerging Trends in Soft Set Theory and Related Topics

**DOI:** 10.1155/2015/689457

**Published:** 2015-01-28

**Authors:** Feng Feng, Naim Cagman, Violeta Leoreanu-Fotea, Muhammad Akram

**Affiliations:** ^1^Department of Applied Mathematics, School of Science, Xi'an University of Posts and Telecommunications, Xi'an 710121, China; ^2^Department of Mathematics, Faculty of Arts and Sciences, University of Gaziosmanpasa, 60100 Tokat, Turkey; ^3^Faculty of Mathematics, Alexandru Ioan Cuza University, 700506 Iasi, Romania; ^4^College of Information Technology, University of the Punjab, Old Campus, Lahore 54000, Pakistan

Uncertainty is an essential and ubiquitous factor in the real world. The meaning and utility of uncertainty have been pondered by theoreticians and practitioners for more than two millennia. It has been found that the concept of uncertainty is too complicate to be captured within a single framework. In response to this, a number of different approaches including probability theory, fuzzy sets, and rough sets have been developed to cope with uncertainty from distinct angles of views.

Molodtsov's soft set theory was initiated in 1999, which provides us with a new framework for handling uncertainty. The philosophy of soft sets is founded on the idea of parametrization, which is utilized by human to perceive and understand concepts of high complexity in daily life. Soft set theory suggests that complicated concepts with intrinsic uncertainty should be characterized from a variety of different aspects, and all these related facets are supposed to be organized and treated as a whole. Without any restriction on its parameters, this theory comes with an ability to model uncertain concepts in a convenient and meaningful way. As a newly emerging area of interdisciplinary research, soft set theory, has received much attention from researchers all over the world. Evidence of this can be found in the increasing number of high-quality works on soft sets and related topics that have been published over past few years.

This special issue mainly aims to provide the researchers with a collection of state-of-the-art research contributions which represent some emerging trends in soft set theory and related topics. It received an overwhelming response from the community. Editors received 47 submissions from different countries around the world. All submissions followed the same standard as applied to regular submissions to this journal. Due to the limited space, 18 articles were finally selected to be published. Contributions of the included papers are briefly summarized as follows.

In “*Concave soft sets, critical soft points, and union-soft ideals of ordered semigroups,*” Y. B. Jun et al. introduce soft algebraic notions such as union-soft semigroups and union-soft (left, right) ideals by organizing parameters with the structure of an ordered semigroup. They examine some basic aspects of these notions mainly under the regularity condition. In addition, the authors define concave soft sets, critical soft points, union-soft products, and union-soft semiprime soft sets and investigate some related properties as well.

In “*Hesitant fuzzy soft subalgebras and ideals in BCK/BCI-algebras,*” Y. B. Jun et al. apply hesitant fuzzy soft sets to the exploration of BCK/BCI-algebras. They define hesitant fuzzy soft subalgebras and (closed) hesitant fuzzy soft ideals over BCK/BCI-algebras, investigate their algebraic properties, and obtain a number of meaningful results.

In “*Soft mappings space,*” T. Y. Ozturk and S. Bayramov introduce soft compact-open topologies in functional spaces of soft topological spaces. They also ascertain interrelations among several functional spaces with soft compact-open topologies.

In “*Soft translations and soft extensions of BCI/BCK-algebras,*” N. Sultana et al. define soft translations of soft subalgebras and soft ideals over BCI/BCK-algebras. They also introduce a related notion called soft extensions and explore the relations between soft translations and soft extensions.

In “*Soft covering based rough sets and their application,*” Ş. Yüksel et al. generalize Pawlak's rough sets based on covering soft sets. They introduce soft covering approximation spaces and soft covering rough approximation operators. Moreover, they present a practical example involving prostate cancer risk data of 78 patients from Selcuk University Meram Medicine Faculty to illustrate the potential applications of soft covering based rough sets in medicine.

In “*A new approach to entropy and similarity measure of vague soft sets,*” D. Hu et al. focus on uncertainty measures of vague soft sets. They propose a new axiomatic definition of entropy and develop a new method to construct the similarity measures and entropies for vague soft sets. They also describe various inner relationships among these uncertainty measures of vague soft sets.

In “*Cyclic soft groups and their applications on groups,*” H. Aktaş and Ş. Özlü define orders of soft groups, powers of soft sets, and cyclic soft groups. They investigate some algebraic properties of cyclic soft groups and reveal the relationships between cyclic soft groups and classical groups.

In “*Ideal theory in semigroups based on intersectional soft sets,*” S. Z. Song et al. introduce int-soft semigroups, int-soft (left, right, and two-sided) ideals, and int-soft quasi-ideals by equipping the parameter set with an associative operation. They present some characterizations of subsemigroups, left ideals, and right ideals based on these notions. They characterize int-soft semigroups and int-soft (left, right, and two-sided) ideals by virtue of int-soft products. Moreover, the authors also discuss the characterization of regular semigroups using int-soft quasi-ideals.

In “*On soft *β*-open sets and soft *β*-continuous functions,*” M. Akdag and A. Ozkan present the concepts of soft *β*-interiors and soft *β*-closures of soft sets in soft topological spaces. They also investigate some properties of soft *β*-open sets and soft *β*-continuous functions.

In “*Novel applications of intuitionistic fuzzy digraphs in decision support systems,*” M. Akram et al. present several decision support models based on intuitionistic fuzzy digraphs. In particular, they describe potential applications of intuitionistic fuzzy digraphs in organization management, medical diagnosis, marketability of books, and vulnerability assessment of gas pipeline networks. They also design and implement the algorithms for these decision support models.

In “*Similarity measure and entropy of fuzzy soft sets,*” Z. Liu et al. discuss a general approach to similarity measures and entropies of fuzzy soft sets based on fuzzy equivalences. They also compare their method with several existing proposals in the literatures.

In “*Subalgebras of BCK/BCI-algebras based on cubic soft sets,*” G. Muhiuddin et al. apply cubic soft sets to the study of BCK/BCI-algebras. They introduce several basic operations of cubic soft sets. An example is presented to show that the *R*-union of two internal cubic soft sets might not be internal. They also discuss the condition under which the *R*-union of two internal cubic soft sets results in an internal cubic soft set. Basic algebraic properties of cubic soft subalgebras of BCK/BCI-algebras are examined as well.

In “*Type-II fuzzy decision support system for fertilizer,*” A. Ashraf et al. demonstrate the application of type-II fuzzy sets in developing a decision support system for modelling a spatial surface of fertilizer. They present a basic platform for the development of spatial surfaces using a type-II fuzzy inference engine based on human linguistic values.

In “*On topological structures of fuzzy parametrized soft sets,*” S. Atmaca and İ. Zorlutuna explore the topological structure of fuzzy parametrized soft sets and fuzzy parametrized soft mappings. They introduce the notion of quasi-coincidence for fuzzy parametrized soft sets and investigate some basic concepts including closures, interiors, bases, compactness, and continuity in fuzzy parametrized soft topological spaces.

In “*Soft congruence relations over rings,*” X. Xin and W. Li introduce soft congruence relations with regard to rings. They also define soft quotient rings and generalized soft ideals. A one-to-one correspondence between soft congruence relations and idealistic soft rings (or soft ideals) is presented. In addition, the authors establish various soft isomorphism theorems regarding soft rings.

In “*Hesitant fuzzy soft sets with application in multicriteria group decision making problems,*” J. Wang et al. define some operations of hesitant fuzzy soft sets by virtue of Archimedean t-norms and t-conorms. They present some aggregation operators, including HFSWA, HFSWG, GHFSWA, and GHFSWG operators, which are used to develop a multicriteria group decision making approach based on hesitant fuzzy soft sets. The authors also illustrate the newly proposed notions and method with a numerical example.

Finally, in “*Some properties of fuzzy soft proximity spaces,*” İ. Demir and O. B. Özbakır explore some properties of fuzzy soft proximity spaces in Katsaras's sense. They demonstrate that each fuzzy soft proximity could induce a fuzzy soft topology by virtue of fuzzy soft closure operators. They present the notion of fuzzy soft *δ*-neighborhoods as an alternative description of fuzzy soft proximities. Moreover, the authors introduce products of fuzzy soft proximity spaces and analyze the relationship between fuzzy soft proximities and proximities.

We sincerely hope the readers will find this special issue informative and inspiring.

